# A novel multimodal imaging approach for working diagnosis of acute myocardial infarction with non-obstructive coronary arteries: a promising diagnostic strategy

**DOI:** 10.3389/fcvm.2025.1646418

**Published:** 2026-01-09

**Authors:** Giovanni Taverna, Lisa Canton, Lorenza Zilio, Vincenzo Calabrese, Annagrazia Cecere, Maria Teresa Savo, Marco Previtero, Giulia Mattesi, Valeria Pergola, Stefano Da Pozzo, Simone Corradin, Angela Susana, Antonella Cecchetto, Anna Baritussio, Alberto Cipriani, Raffaella Motta, Giuseppe Andò, Gianluca Pontone, Fabrizio Ricci, Carmine Pizzi, Domenico Corrado, Giorgio De Conti, Martina Perazzolo Marra

**Affiliations:** 1 Department of Cardio-Thoraco-Vascular Sciences and Public Health, University of Padua, Padua, Italy; 2 Department of Translational Medicine, Section of Radiology, University of Ferrara, Ferrara, Italy; 3 Department of Medical and Surgical Sciences, Alma Mater Studiorum, University of Bologna, Italy; 4 Cardiovascular Division, Morgagni-Pierantoni University Hospital, Forlì, Italy; 5 Department of Medicine and Surgery, University of Enna, Enna, Italy; 6 Radiology Unit, Azienda Ospedale-Università Padova, Padova, Italy; 7 Department of Clinical and Experimental Medicine, University of Messina, Messina, Italy; 8 Department of Perioperative Cardiology and Cardiovascular Imaging, Centro Cardiologico Monzino IRCCS, Milan, Italy; 9 Department of Biomedical, Surgical and Dental Sciences, University of Milan, Milan, Italy; 10 Department of Neuroscience, Imaging and Clinical Sciences, "G. d'Annunzio" University of Chieti-Pescara, Chieti, Italy; 11 Heart Department, SS. Annunziata Hospital, University Cardiology Division, Chieti, Italy; 12 Institute for Advanced Biomedical Technologies, G. D'Annunzio University of Chieti-Pescara, Chieti, Italy; 13 Department of Clinical Sciences, Lund University, Malmö, Sweden

**Keywords:** CCTA, CMR, high-risk plaque, LIE, MINOCA, troponin-positive chest pain

## Abstract

**Background:**

Myocardial infarction with non-obstructive coronary arteries (MINOCA) demands prompt mechanistic clarification. Early integration of coronary CT angiography (CCTA) and cardiovascular magnetic resonance (CMR) can refine diagnosis during the acute phase.

**Methods:**

Twenty-one consecutive patients (41 ± 10 years; 71% men) presenting with troponin-positive chest pain and unobstructed coronaries underwent CCTA, delayed iodine-enhanced CT for late iodine enhancement (LIE), and CMR imaging within 14 days, with a mean interval of 5 days [interquartile range (IQR) 2–9] between both imaging modalities. CCTA assessed luminal stenosis and high-risk plaque; LIE mapped iodine retention; CMR evaluated myocardial edema and late gadolinium enhancement (LGE). Clinical, electrocardiographic, and laboratory data were collected.

**Results:**

Eight patients were classified as MINOCA and 13 as acute myocarditis. Chest pain was universal; dyspnea and syncope occurred in seven and two patients, respectively. Median peak high-sensitivity troponin-I was 1,569 ng/L (IQR 589–5 771). Biventricular systolic function was preserved (mean LVEF 58%; RVEF 55%). LGE appeared in 16 subjects: subendocardial in every MINOCA case and intramural or subepicardial in eight myocarditis cases. Myocardial edema was present in 15 patients. CCTA showed no atherosclerosis in 16 patients; five displayed non-obstructive lesions (<50% stenosis) with high-risk plaque confined to three MINOCA subjects. LIE confirmed iodine uptake matching the LGE pattern in all MINOCA patients and in six with myocarditis.

**Conclusions:**

An acute CCTA-CMR protocol may aid in distinguishing ischemic from non-ischemic myocardial injury in presumed MINOCA and unmasks occult high-risk plaques. This multimodal imaging approach reveals occult high-risk coronary plaques and enhances diagnostic accuracy, thereby supporting mechanism-targeted management strategies in patients presenting with troponin-positive chest pain.

## 1 Introduction

Myocardial infarction with non-obstructive coronary arteries (MINOCA) is defined by the Fourth Universal Definition of Myocardial Infarction as an acute myocardial infarction without angiographic evidence of obstructive coronary artery disease (>50% stenosis) in a major epicardial vessel, after exclusion of other non-ischemic causes of myocardial injury (1, 2). Clinical presentation accounts for approximately 5%–6% of all acute coronary syndromes (3, 4), with reported prevalence varying between 1% and 13% among patients presenting with myocardial infarction (5–7).

MINOCA remains a working diagnosis in cardiology, encompassing a broad range of underlying mechanisms that necessitate careful differentiation (8). Current ESC and AHA guidelines recommend an initial invasive coronary angiography to exclude obstructive coronary artery disease in MINOCA patients, followed by cardiac magnetic resonance imaging (CMR) for myocardial tissue characterization to differentiate ischemic from non-ischemic injury. In this context, early cardiac magnetic resonance (CMR), performed within 7–14 days of the acute event, plays a crucial role through the detection of characteristic scar patterns and myocardial edema, thereby refining diagnostic accuracy (3, 9, 10). In patients with MINOCA, cardiac magnetic resonance imaging (CMR) has demonstrated substantial diagnostic and prognostic value, proving essential not only for confirming the diagnosis but also for reclassifying approximately 68% of patients initially diagnosed with MINOCA, thereby refining clinical management, and guiding tailored therapeutic strategies (11). Although CMR with late gadolinium enhancement (LGE) remains the reference standard for non-invasive myocardial tissue characterization, its immediate availability in acute clinical settings is often limited and could delay diagnosis. Our proposed protocol, combining early coronary computed tomography angiography (CCTA) with late iodine enhancement (LIE) and subsequent CMR, offers a rapid, non-invasive multimodal approach that aligns with these guidelines. It supports early differentiation of myocardial injury type and identification of high-risk plaques, potentially optimizing patient stratification and guiding tailored management when CMR access is constrained. Coronary computed tomography angiography (CCTA) offers a faster alternative, providing comprehensive evaluation of coronary anatomy and atherosclerotic plaque morphology, including features of plaque vulnerability such as low attenuation, positive remodeling, spotty calcifications, and the napkin-ring sign.

More recently, the application of late iodine enhancement (LIE) techniques in cardiac CT has enabled the detection of myocardial fibrosis, exploiting the delayed kinetics of iodinated contrast agents, which parallel those of gadolinium-based contrast on CMR. Areas of increased iodine concentration within scar tissue can thus be visualized 10–15 min after contrast administration, offering an additional layer of tissue characterization (12).

The aim of this study was to evaluate the diagnostic role of an integrated noninvasive multimodal imaging approach, combining early CCTA and CMR, in patients presenting with suspected MINOCA.

## 2 Methods

### 2.1 Study design and population

We retrospectively screened 750 consecutive adults (≥18 years) admitted with troponin-positive acute chest pain between December 2022 and December 2024 at the University Hospital of Padua and S. Orsola-Malpighi Hospital. A working diagnosis of acute myocardial infarction with non-obstructive coronary arteries (MINOCA) was suspected after exclusion of alternative non-ischemic triggers of biomarker release (e.g., sepsis, stroke, severe anemia). All included patients underwent coronary CT angiography (CCTA) and cardiovascular magnetic resonance (CMR) within 14 days of presentation to enable early etiological classification. The present study was conducted according to the Declaration of Helsinki and all patients gave written informed consent for use of their clinical data. Data collection, analysis, publication, and storage were approved by the local medical ethics committee.

### 2.2 Inclusion and exclusion criteria

**Figure 1:**
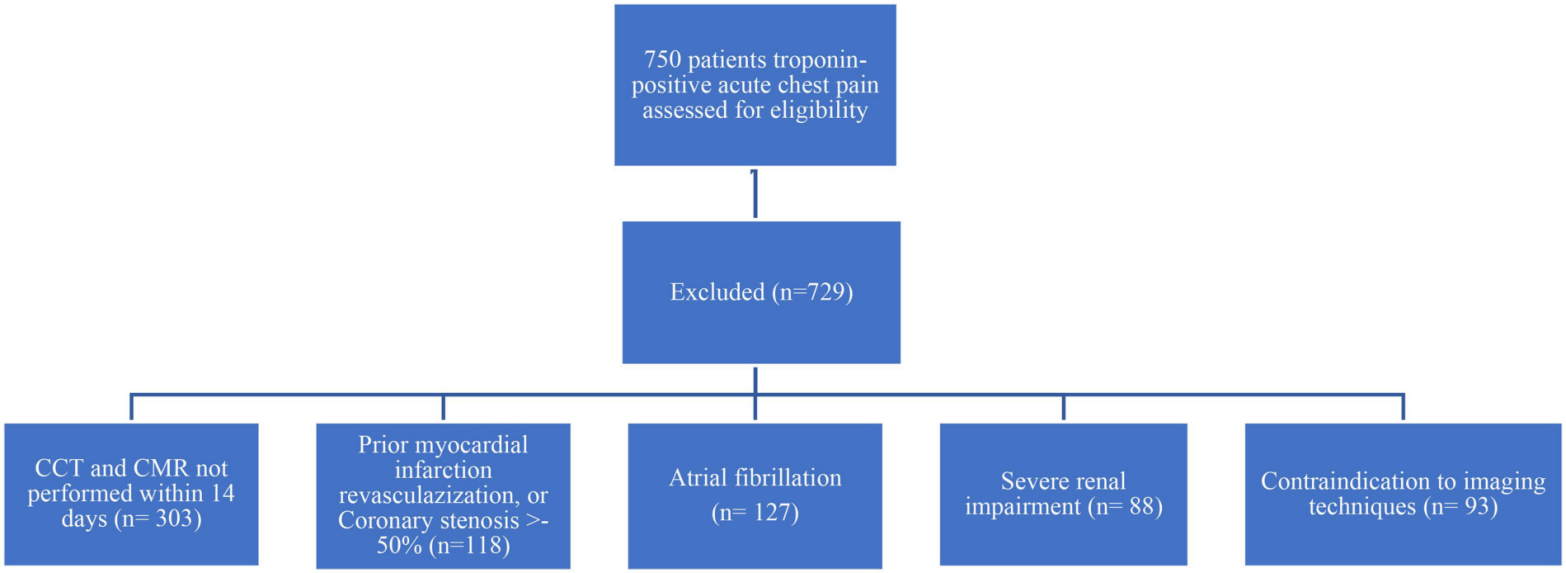
Patient screening and exclusion criteria flow chart diagram.

Eligibility required (i) clinical suspicion of acute myocardial injury with non-obstructive coronaries, (ii) paired CCTA and CMR—including parametric mapping—within 14 days, and (iii) complete clinical and laboratory data. Prior myocardial infarction or revascularization, coronary stenosis ≥50%, atrial fibrillation, severe renal impairment (eGFR <30 mL min^−1^ 1.73 m^−2^), or any absolute contraindication to CCTA/CMR prompted exclusion. Patients with atrial fibrillation were excluded to reduce the risk of image quality problems caused by irregular heartbeats, which can affect the accuracy of both CCTA and CMR scans. Irregular rhythm could be the cause of motion artifacts and reduce image clarity, interfering with the detailed assessment of the myocardium. The flow chart diagram visually summarizes this screening and exclusion process (Figure 1). Twenty-one patients met all criteria: 13 satisfied the 2013 ESC myocarditis criteria (13), and 8 fulfilled the fourth Universal Definition of MINOCA.

### 2.3 Clinical assessment, laboratory tests and echocardiography

At admission all participants underwent structured clinical evaluation, 12-lead ECG, high-sensitivity troponin-I (TnI-HS), C-reactive protein (CRP), and transthoracic echocardiography.

Images were acquired on Philips iE33 or GE Vivid 7/9 platforms using standard parasternal long- and short-axis and apical two-, three-, and four-chamber views (three consecutive cardiac cycles). Two cardiologists with >10 years’ experience analyzed studies offline.

### 2.4 CCTA acquisition and late iodine enhancement (LIE) scanning

Examinations were performed on a 320-row scanner (Aquilion ONE, Canon Medical Systems) using prospective ECG-gated acquisition during breath hold. (0.5 mm collimation). Intravenous metoprolol (5–40 mg) was administered to achieve heart rate <65 bpm; 0.5 mg sublingual nitroglycerin optimized coronary caliber. This preparation helps optimize image quality during coronary CT angiography. In contrast, the CMR protocol did not involve such pharmacologic preparation. The tube voltage was set between 100 and 130 kVp, with a temporal resolution of 175 ms. The tube current was adaptively modulated (SUREexposure, Toshiba Medical System, Japan) and included ECG-based tube current modulation to reduce radiation dose. Image acquisition consisted of an early arterial phase, triggered using real-time bolus tracking (Sure Start, Toshiba Medical Systems, Japan) with a region of interest placed in the descending thoracic aorta at mid-heart level. A 50–70 mL bolus of non-ionic iodinated contrast (Iomeprol, 400 mg iodine/mL, Bracco Imaging S.p.A) was injected through an 18-gauge intravenous cannula using a power injector (Medrad Stellant CT System, Bayer Healthcare) at 5 mL/s, immediately followed by a 40 mL saline flush. Scanning was automatically initiated when enhancement in the ROI reached 180 Hounsfield units. Images were reconstructed with a slice thickness of 0.5 mm and an increment of 0.25 mm using a standard soft-tissue reconstruction kernel. LIE images were obtained 7–10 min post-contrast to differentiate normal from injured myocardium. Post-processing employed volume-rendered and curved-multiplanar reformations with vessel centerline analysis.

For accurate and comprehensive interpretation of myocardial images, high-quality reconstruction of the left ventricular short- and long-axis views was performed. During visual assessment, reconstructed images were typically viewed at a slice thickness of approximately 5–8 mm, which striked an optimal balance between preserving image detail and reducing noise, thereby enhancing diagnostic clarity (13).

### 2.5 Plaque analysis

A validated semi-automated platform (QAngioCT Research Edition v3.2.14.4, Medis) quantified plaque components within manually defined segments. High-risk plaque (HRP) features evaluated were positive remodeling, low-attenuation core, spotty calcification, and napkin-ring sign. Two independent observers performed all measurements; discrepancies were resolved by consensus. A third reader was not involved in resolving disagreements. This method helped maintain consistency and reliability in plaque evaluation.

### 2.6 CMR protocol

All scans were acquired on a 1.5T system (Magnetom Avanto, Siemens Healthcare) with a dedicated protocol, including spin-echo and postinjection sequence contrast. Cine steady-state free-precession sequences covered the ventricles in contiguous short-axis slices (6 mm thickness, 0 mm gap) and standard long-axis planes. Imaging parameters included repetition times ranging from 2.5 to 3.8 ms, echo times between 1.1 and 1.6 ms, spatial resolutions ranging from 2.0 to 2.8 mm in-plane, and flip angles between 45° and 60°. The temporal resolution was maintained between 30 and 40 ms. Additionally, long-axis views were obtained in 2–4 standard planes to provide comprehensive assessment. Right ventricular function was evaluated using transaxial balanced SSFP cine images, acquired from the right ventricular outflow tract down to the diaphragm, employing at two-chamber projection. Left ventricular function assessment was also performed using the same cine SSFP sequences on the short-axis and long-axis planes, ensuring both chambers were accurately evaluated. Ventricular volumes were measured and indexed to the patient's body surface area, ensuring standardized comparisons across subjects. T1-weighted turbo spin-echo sequences were acquired in the axial plane to assess for myocardial fat infiltration. In selected cases, T2-weighted short tau inversion recovery (STIR) images were acquired to confirm the presence of intramyocardial fat. Myocardial edema was assessed with T2-weighted short-tau inversion recovery (STIR) imaging [signal intensity >2 SD vs. skeletal muscle (14)]. Native T1/T2 (normal reference values T1 970 ± 70 ms, T2 49 ± 5 ms) and extracellular-volume mapping were obtained in basal, mid-ventricular, and apical short-axis positions. Late gadolinium enhancement (LGE) images were acquired 10–15 min after 0.2 mmol kg^−1^ gadobenate-dimeglumine (Multihance, Bracco) using phase-sensitive inversion recovery with inversion time individually adjusted to null normal myocardium and maximize contrast between healthy and diseased tissue. For LGE, a threshold of five standard deviations above normal myocardial signal intensity was applied (15). Analyses were performed on a macOS analysis program CVI 42 software (Circle Cardiovascular Imaging) and the presence, pattern, and location of LGE were reviewed on axial, short-axis, and long-axis images by observers blinded to clinical data to avoid bias. CMR image analysis segment by segment was done without access to the CCTA and LIE results, and similarly, the CCTA and LIE images were reviewed without knowledge of the CMR findings. This procedure was followed to reduce interpretation bias and maintain the objectivity of the assessments.

### 2.7 Statistical analysis

Continuous data are presented as mean ± SD or median (interquartile range) according to distribution (Kolmogorov–Smirnov test and visual inspection). Categorical variables are expressed as counts and percentages. Differences in complete concordance between imaging findings and final diagnosis (MINOCA vs. myocarditis) were evaluated with *χ*^2^ or Fisher's exact test, as appropriate. Spearman correlation assessed the association between concordance and disease category. Analyses were conducted in R (v4.4.1); two-tailed *p* < 0.05 denoted significance.

## 3 Results

Twenty-one consecutive patients with unobstructed coronary arteries at invasive angiography were studied (mean age, 41 ± 10 years; 15 men). At discharge, 13 patients met the ESC criteria for acute myocarditis and 8 fulfilled the Fourth Universal Definition of MINOCA. Chest pain was universal; dyspnea preceded presentation in 7 patients and syncope in 2. Nine patients described a febrile illness within the preceding 2 weeks, and 2 reported recent recreational drug use. Electrocardiography revealed diffuse ST-segment elevation in 4 patients, isolated ST-segment depression in 1, hyperacute T waves in 7, and T-wave inversion in 4. Median peak high-sensitivity troponin I was 1,569 ng per liter (IQR, 589–5,771); the median C-reactive protein level was 1.38 mg per deciliter (IQR, 0.10–4.90). Estimated glomerular filtration rate exceeded 60 ml per minute per 1.73 m^2^ in every patient. Transthoracic echocardiography showed a mean left-ventricular ejection fraction of 55 ± 4 percent with no regional wall-motion abnormalities. All included patients underwent coronary CT angiography (CCTA) and cardiovascular magnetic resonance (CMR) within 14 days of acute presentation, with a mean interval of 5 days (IQR 2–9). The mean interval was 3 days (IQR 2–6) for MINOCA and 7 days (IQR 2–9) for myocarditis, with no significant difference between subgroups (*p* = 0.36). Cardiovascular magnetic resonance imaging demonstrated preserved or mildly reduced left-ventricular ejection fraction (58 ± 2 percent in MINOCA and 55 ± 6 percent in myocarditis) and normal right-ventricular function (55 ± 1 percent and 53 ± 2 percent, respectively). Myocardial edema was present in 15 patients. Late gadolinium enhancement (LGE) was identified in 16 patients and was subendocardial in every MINOCA case, whereas it was intramural or subepicardial in 8 myocarditis cases. In each MINOCA patient, the infarct-related artery inferred from the LGE distribution corresponded to a coronary segment on computed tomography.

Cardiovascular risk factors, laboratory findings, ECG at the admission and CMR features split for myocarditis (13) and MINOCA (8) are summarized in Tables 1, 2.

**Table 1:** Cardiovascular risk factors, laboratory findings and ECG at the hospital admission.

Variable	Myocarditis (13)	Minoca (8)	*P*-value
Age (years)	32 ± 20	50 ± 13	0.305
Sex, *n* female (%)	1 (7)	5 (62)	0.007
Family history of CAD	5	2	0.525
Smokers	6	4	0.864
Hypertension	1	4	0.027
Dyslipidemia	1	2	0.271
Previous TIA/ictus	2	0	0.243
Peak hs-Troponin I level, ng/L	2009 [485–11,789]	1,144 [667–2,895]	0.834
Peak C reactive protein level, mg/dL	4.5 [2.4–5.8]	0.06 [0.06–0.29]	<0.001
eGFR, mL min^−1^ 1.73 m^−2^	111 [95–118]	87 [69–103]	0.109
ST elevation	4	0	0.006
ST depression	0	1	0.598
Hyperacute T-waves	6	1	0.088
Negative T-waves	2	2	0.191
Q necrosis	0	1	0.586

CAD, coronary artery disease; eGFR, estimated glomerular filtration rate.

**Table 2:** Morphological, functional and tissue CMR features.

Variable	Myocarditis (13)	Minoca (8)	*P*-value
LV EDVi (mL/mq)	90 ± 12	79 ± 15	0.256
LV ESVi (mL/mq)	38 ± 4	37 ± 6	0.834
LVEF (%)	58 ± 2	55 ± 6	0.173
RV EDVi (mL/mq)	91 ± 20	75 ± 15	0.173
R V ESVi (mL/mq)	45 ± 11	37 ± 6	0.057
RVEF (%)	55 ± 1	53 ± 2	0.698
T1 mapping, ms	1,001 [991–1,015]	982 [958–1,025]	0.63
T2 mapping, ms	49 [48–55]	47 [46–49]	0.12
Myocardial edema	7	8	0.023
Myocardial LGE	8	8	0.044

LV EDVi, left ventricular end-diastolic volume index; LV ESVi, left ventricular end-systolic volume index; LVEF, left ventricular ejection fraction; RV EDVi, right ventricular end-diastolic volume; RV ESVi, right ventricular end-systolic volume; RVEF, right ventricular ejection fraction.

**Figure 2:**
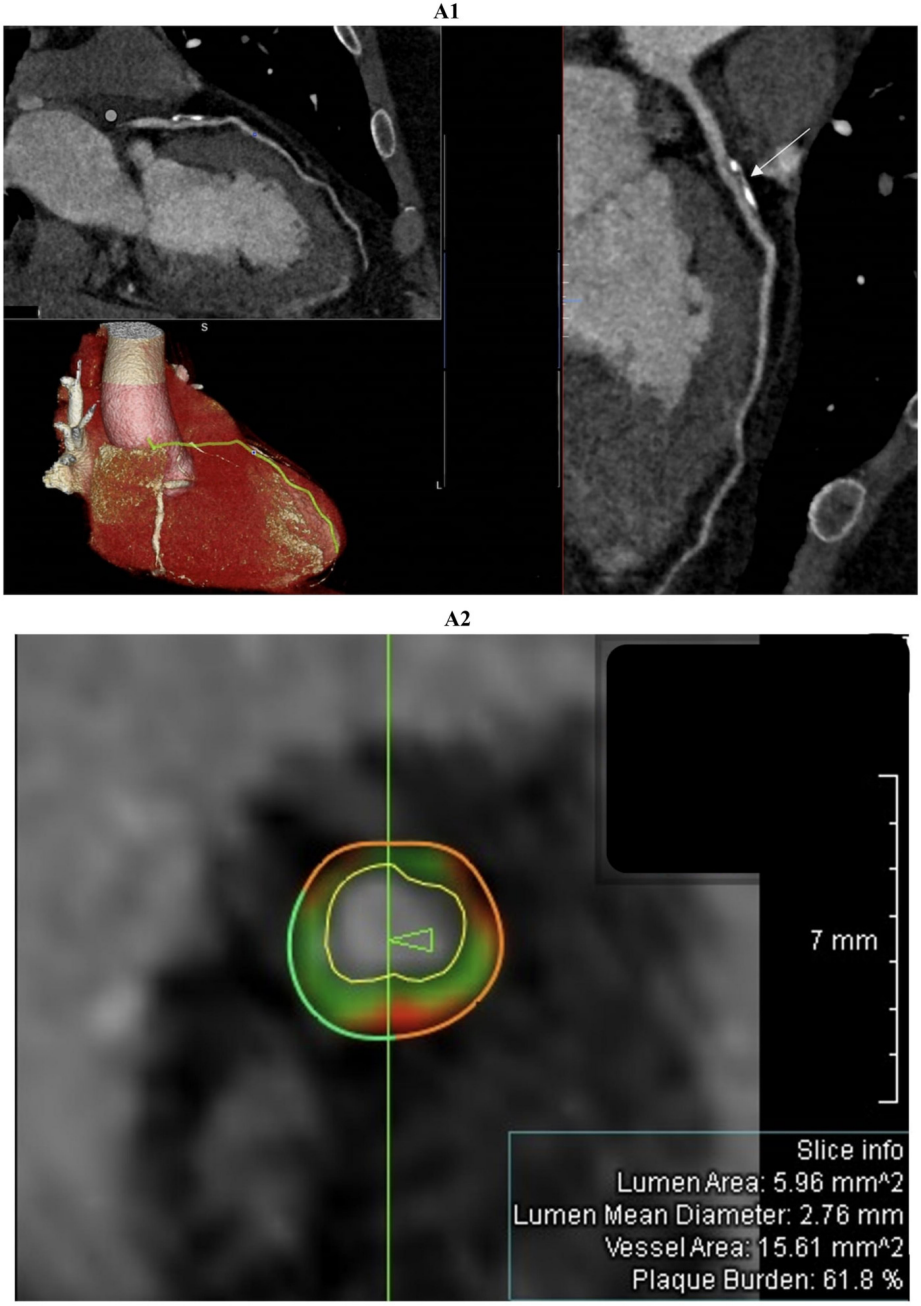
Comprehensive coronary artery evaluation using coronary computed tomography angiography (CCTA) with advanced image reconstructions and quantitative plaque analysis was performed in a 51-year-old male patient admitted with suspected acute myocardial infarction with non-obstructive coronary arteries (MINOCA). The curved multiplanar reconstruction (figure 2A1, top left panel and large right panel) offers a detailed longitudinal view of the left anterior descending (LAD) artery, clearly delineating the vessel lumen and wall morphology. The proximal LAD segment reveals an extensive, eccentrically located plaque predominantly composed of calcific tissue. This lesion exhibits typical features of positive remodeling without causing significant luminal narrowing or flow limitation. The three-dimensional volume-rendered image (figure 2A1, bottom left panel) shows the heart and coronary arteries with the LAD artery highlighted in yellow, facilitating spatial comprehension of plaque localization and vessel trajectory from the left main coronary artery to the apex. Cross-sectional imaging (figure 2A2), indicated by the white arrow in figure 2A1, combined with quantitative coronary plaque analysis identifies a high-risk plaque with positive remodeling. The green vertical line marks the exact location of the cross-section shown in figure 2A2 and the arrow indicates the slice orientation relative to the vessel. This concentric plaque includes dark green fibrous tissue, light green fibrofatty tissue, and red necrotic core, which are characteristic of high-risk features. The external vessel wall contour is partly dark green, representing fibrous tissue, and partly red, indicating the necrotic core. The internal vessel wall contour, shown in light green, corresponds to fibrofatty tissue. The white boundary inside the vessel marks the lumen contour, defining the blood flow space. Lumen area (5.96 mm^2^), mean diameter (2.76 mm), vessel area (15.61 mm^2^), and plaque burden (61.8%) are reported in the slice info box.

Coronary CT angiography was performed following invasive coronary angiography that excluded stenosis greater than 50%, as the patients continued to experience chest pain and still demonstrated abnormal findings on their electrocardiogram (ECG). CCTA showed no atherosclerosis in 16 patients; the remaining 5 (including 3 with MINOCA) had non-obstructive (<50 percent) lesions in the left anterior descending artery, often accompanied by minor irregularities in the circumflex or right coronary arteries. High-risk plaque features—positive remodeling, low-attenuation core, spotty calcification, or napkin-ring sign—were present in 3 MINOCA patients (Figure 2). Late iodine enhancement (LIE) performed 7−10 min after contrast administration most frequently revealed subepicardial or mid-wall iodine uptake; subendocardial enhancement, primarily in the territory of the left anterior descending artery (specifically the basal anterior interventricular septum and the basal anterior wall of the left ventricle) was observed in the same 8 patients with MINOCA.

**Figure 3:**
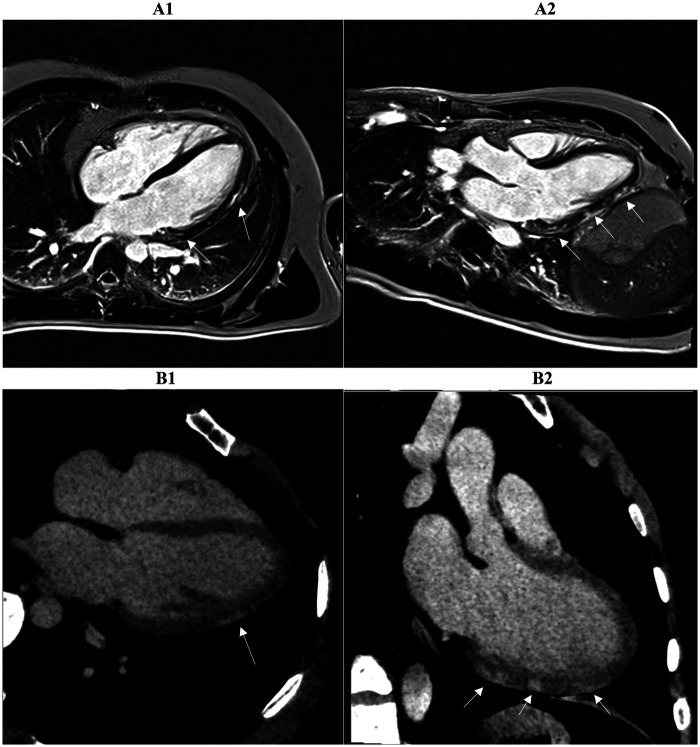
Two myocardial tissue characterization slices on CMR (A1, A2) and CCTA (B1, B2) in the same patient diagnosed with myocarditis at discharge are shown. Both the long-axis four-chamber and three-chamber views reveal intramural or sub-epicardial enhancement (arrows) with 80% segmental concordance between the imaging techniques, based on matching presence or absence of enhancement in corresponding myocardial regions. CMR, cardiac magnetic resonance; CCTA, coronary computed tomography angiography.

**Figure 4:**
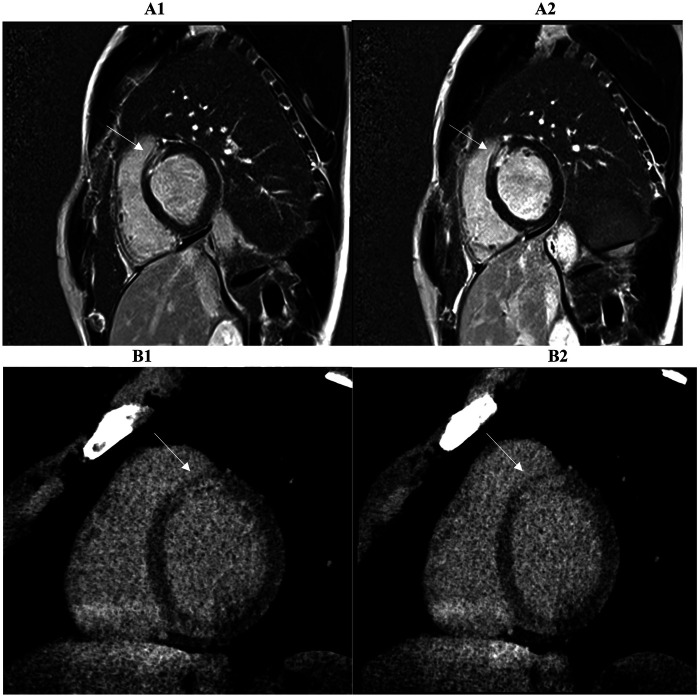
Two consecutive basal and mid-basal short-axis PSIR (phase sensitive inversion recovery) images (A1, A2) demonstrate transmural late gadolinium enhancement (LGE) in the territory of the anterior descending coronary artery (basal antero-septal wall, arrow) in a patient with suspected myocardial infarction with non-obstructed coronary arteries (MINOCA). The corresponding prior late iodine enhancement (LIE) images (B1, B2) show involvement of the same myocardial segments. The two imaging modalities exhibit 100% segmental concordance, defined as complete agreement on the presence or absence of enhancement within matching myocardial segments.

**Figure 5:**
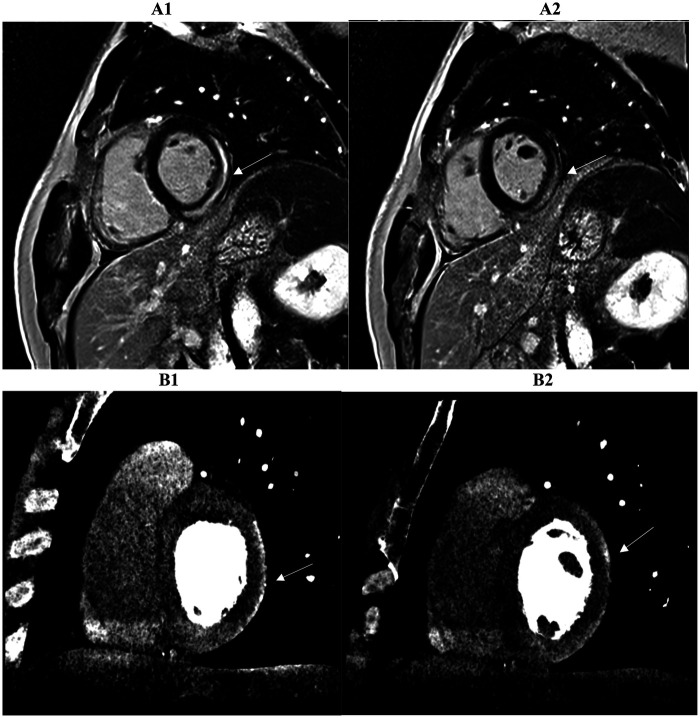
Two consecutive basal and mid-basal short-axis images demonstrate a typical subepicardial scar of the inferolateral wall highlighted by late iodine enhancement (LIE) (B1, B2). The CCTA findings suggested myocarditis and were confirmed by cardiac MRI performed within 14 days of the acute event. A subepicardial-intramural stria of late gadolinium enhancement (LGE) is seen on the basal and mid-basal short-axis LGE images (A1, A2), with 83% spatial agreement between the two techniques based on the matching presence or absence of myocardial enhancement in the corresponding segments (arrow).

On a segment-by-segment comparison (Figure 3), LIE and LGE findings agreed in 17 of 21 patients (81 percent). Complete segmental concordance was present in all 8 MINOCA cases (Figure 4) and in 2 myocarditis cases. In the remaining myocarditis patients, concordance ranged from 25 to 83 percent of affected segments (Figure 5). Overall agreement (Table 3) was 100 percent in MINOCA and 15 percent in myocarditis (*P* < 0.001). Subendocardial enhancement correlated strongly with a final diagnosis of MINOCA (Spearman's *ρ* = 0.82, *P* < 0.001).

**Table 3:** Per-patient imaging findings and concordance levels between LIE and LGE.

Patient ID	Diagnosis	LIE Pattern	LGE Pattern	Segmental concordance (%)
1	Myocarditis	Mid-wall/Subepicardial	Mid-wall/Subepicardial	0
2	Myocarditis	Mid-wall/Subepicardial	Mid-wall/Subepicardial	25
3	Myocarditis	Mid-wall/Subepicardial	Mid-wall/Subepicardial	83
4	Myocarditis	Mid-wall/Subepicardial	Mid-wall/Subepicardial	100
5	MINOCA	Subendocardial	Subendocardial	100
6	Myocarditis	Mid-wall/Subepicardial	Mid-wall/Subepicardial	50
7	Myocarditis	Mid-wall/Subepicardial	Mid-wall/Subepicardial	50
8	Myocarditis	Mid-wall/Subepicardial	Mid-wall/Subepicardial	0
9	Myocarditis	Mid-wall/Subepicardial	Mid-wall/Subepicardial	100
10	Myocarditis	Mid-wall/Subepicardial	Mid-wall/Subepicardial	80
11	Myocarditis	Mid-wall/Subepicardial	Mid-wall/Subepicardial	0
12	MINOCA	Subendocardial	Subendocardial	100
13	Myocarditis	Mid-wall/Subepicardial	Mid-wall/Subepicardial	0
14	Myocarditis	Mid-wall/Subepicardial	Mid-wall/Subepicardial	50
15	MINOCA	Subendocardial	Subendocardial	100
16	Myocarditis	Mid-wall/Subepicardial	Mid-wall/Subepicardial	67
17	MINOCA	Subendocardial	Subendocardial	100
18	MINOCA	Subendocardial	Subendocardial	100
19	MINOCA	Subendocardial	Subendocardial	100
20	MINOCA	Subendocardial	Subendocardial	100
21	MINOCA	Subendocardial	Subendocardial	100

The median effective radiation dose delivered during the combined CCTA and LIE protocol was 8.78 millisieverts (interquartile range, 5.54–10.29 millisieverts). The volume of iodine contrast administered had a median value of 54.0 milliliters, ranging from 50 to 70 milliliters. These figures fall within accepted safety standards for diagnostic imaging, supporting the protocol's suitability in terms of patient radiation exposure and contrast use. Efforts to further optimize these parameters to minimize risks while preserving image quality are ongoing.

During one-year follow-up, only four myocarditis patients (30%) were readmitted with recurrent chest pain and diffuse ST-segment elevation; repeat imaging confirmed chronic inflammatory injury. No MINOCA patient experienced recurrent events, MACE, rehospitalizations or deaths in the same period.

## 4 Discussion

The present study shows that a single comprehensive CT protocol, integrating coronary imaging, quantitative plaque analysis, and LIE, accurately distinguishes ischemic from inflammatory myocardial injury in patients presenting with troponin-positive chest pain and unobstructed coronary arteries. Subendocardial LIE in the presence of high-risk plaque features consistently identified patients with MINOCA, whereas a mid-wall or subepicardial enhancement pattern in the absence of high-risk plaque was typical of myocarditis. Segment-by-segment analysis demonstrated complete concordance between LIE and LGE in MINOCA, while agreement was significantly lower in myocarditis, reflecting the structural heterogeneity and temporal dynamics of inflammatory injury. These findings support the integration of a multimodal CT approach early in the diagnostic pathway, allowing simultaneous assessment of coronary anatomy, plaque characteristics, and myocardial tissue status. An ischemic pattern on CT enables immediate initiation of mechanism-targeted therapies, whereas a non-ischemic pattern shifts clinical focus towards inflammatory or alternative causes, reducing diagnostic uncertainty. CMR retains its essential role as the reference standard for detailed tissue characterization, functional quantification, and follow-up evaluation, reinforcing the diagnostic framework established by early CT.

This study offers new insights into the potential of multimodal imaging for diagnosing MINOCA and differentiating it from myocarditis, a common diagnostic challenge. The integration of CCTA and CMR allowed for precise identification of myocardial injury patterns, potentially guiding more accurate diagnosis and therapeutic decisions in patients presenting with acute chest pain and elevated troponin levels. Troponin-positive chest pain with unobstructed coronary arteries indeed represents a daily diagnostic dilemma, and the research of the underlying pathophysiological mechanism is of priority importance for improving the management and the subsequent treat­ment. For this reason, cardiac MRI plays a crucial role providing a diagnosis in many cases but not in all. On the other hand, the assessment of MINOCA patients using early CCTA helps to understand the invisible coronary mechanisms involved in myocardial damage, including the quantitative and qualitative analysis of coronary plaque, and the assessment of other novel parameters such as the late iodine enhancement (LIE). This novel parameter is supported by many studies as a surrogate marker of fibrosis but in comparison to LIE, LGE has a higher contrast resolution (16). This is likely due to the inherently superior contrast resolution of gadolinium-based MRI. In our study, according to Palmisano et al. (17), a faster administration of LIE, for example at the hospital admission, helped us to identify the cause of symptoms and acute myocardial injury in patients with suspected MINOCA. The contemporary use of CMR, performed within 14 days of the acute coronary syndrome, allowed us to confirm or not the previous CCTA results.

In line with the literature, our results confirmed the achievability and credibility of tis­sue characterization at CCTA using LIE to identify the normal or abnormal myocardium (16).

**Figure 6:**
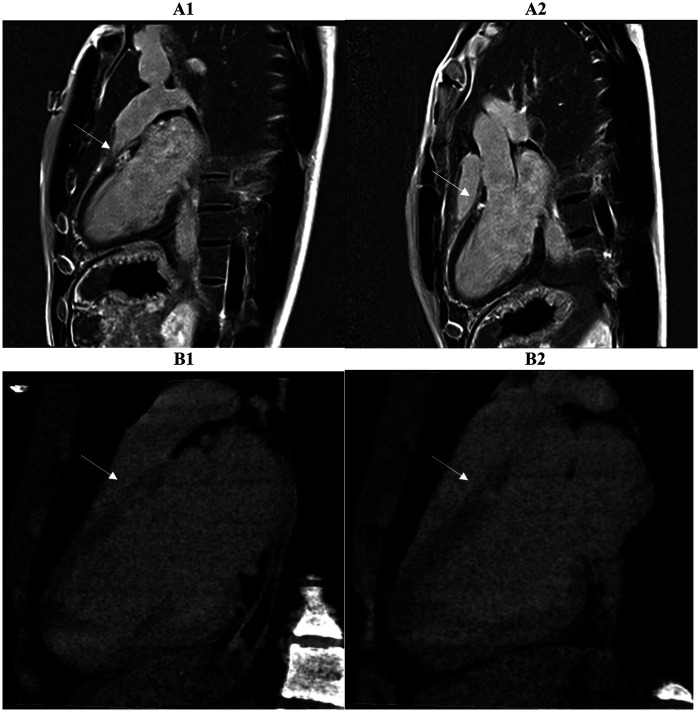
A strong correlation of scar tissue burden is observed between late gadolinium enhancement (LGE) (A1, A2, top row) and late iodine enhancement (LIE) (B1, B2, bottom row) in two- and three-chamber long-axis views. Both imaging methods demonstrated complete concordance (100%) in identifying myocardial segments showing enhancement, indicating full agreement in the detection of scarred tissue in a patient with suspected ischemic cardiomyopathy. Arrows mark the areas involved.

A stronger correlation between the scar tissue burdens measured by LIE and LGE was found in our patients with ischemic cardiomyopathy with an agreement (100%) between both imaging techniques, regardless of the physician's experience level (Figure 6). This strong agreement in identifying the presence or absence of scar tissue felt to 25%–83% for non-ischemic cardiomyopathy scars, like myocarditis, in which CMR remains the gold standard for detailed tissue characterization and diagnostic certainty.

Baudry et al (18). reported that CT could reveal LIE in the subepicardial layer of the inflamed myocardial wall. An increased uptake of contrast agent in injury tissue is synonym of an increased permeability of cellular membranes consistent with active inflammatory process.

In our study we found that LIE compared to LGE has revealed high sensitivity and specificity in diagnosing acute myocarditis and similarly to Bouleti et al. (19), who aimed to compare LIE on spectral CT with the reference LGE in acute myocarditis, the overall accuracy of spectral CT was less of 96%.

Based on our results, LIE in non-ischemic cardiomyopathy dem­onstrated a tendency to underestimate the extension of scar compared with that of LGE, without loss in diagnostic specific­ity and accuracy. In a recent study by Palmisano et al. (17), LIE demonstrated the mild underestimation of the scar extension compared to LGE subepicardial post-myocarditis scars, by both the readers although the impact of readers’ experience. The systematic underestimation of scar burden by LIE in myocarditis likely reflects the evolving permeability and extracellular space associated with active inflammation.

We hypothesize that the underestimation of scar extent may be attributed to the complex and dynamic nature of the inflammatory process. The degree of contrast agent uptake is influenced by cellular membrane permeability, and the distribution of iodine-based delayed enhancement reflects transient tissue injury, which in the acute setting can evolve significantly from day to day. Because myocarditis involves ongoing inflammation that evolves over time, the areas seen on early LIE may look different or even less visible when the CMR is done much later. LIE captures the heart tissue shortly after contrast injection, while CMR done days later reflects the state of the heart as the inflammation changes or starts to heal. This difference in when the images are taken means that what appears as injury on LIE might have improved or changed by the time of the CMR, leading to less overlap between the two imaging methods in myocarditis patients.

Based on the results, early LIE shows promise for myocardial characterization but does not fully replace CMR, especially in cases like myocarditis. The contrast-to-noise ratio (CNR) in CT imaging is lower than that of CMR, and interpretation of LIE requires greater radiologist experience. However, experienced readers can detect myocardial scars with comparable accuracy on both modalities. Despite this, CMR remains the gold standard for myocardial tissue characterization due to its ability to perform quantitative T1 and T2 mapping and LGE, capabilities currently unavailable with CT technology. While LIE can provide valuable early information and may be a useful alternative in centers with limited MRI access, our findings suggest it should remain complementary to CMR rather than a replacement. CMR's ability to capture a broader range of tissue changes at different stages remains crucial for a comprehensive assessment.

Emerging technologies, particularly photon-counting CT, hold promise for improving the sensitivity of myocardial tissue characterization at the CT level, offering higher spatial resolution, superior contrast differentiation, and reduced noise compared to conventional detectors. This technology can more precisely differentiate little differences in myocardial tissue composition and contrast uptake, which is especially valuable in conditions like myocarditis where distinguishing acute inflammation from chronic injury is critical. So, in this setting, photon-counting CT could enhance the detection and characterization of myocardial abnormalities by providing clearer and more detailed images of subendocardial and inflammatory changes, potentially reducing false positives and improving concordance with CMR results. This would allow for more accurate assessment of the timing and extent of myocardial injury. During one-year follow-up, only four myocarditis patients (30%) were readmitted to the hospital with a final diagnosis of chronic inflammatory injury. This 30% readmission rate matches what has been reported in literature (20). It's known that while many patients recover, some continue to have symptoms or recurrent episodes due to ongoing inflammation. Previous research shows that around 20%–40% of myocarditis patients experience similar recurring issues during follow-up (21). This highlights the need for careful monitoring and follow-up in these patients. Although limited by sample size and short-term follow-up, the study provides a coherent and clinically actionable model for integrating CT and CMR in the evaluation of troponin-positive chest pain with unobstructed coronaries. A comprehensive CCTA protocol can serve as a one-stop diagnostic tool in acute chest pain evaluation, enabling assessment of obstructive coronary artery disease, acute aortic syndromes, pulmonary embolism, and myocardial injury via LIE and extracellular volume (ECV) assessment. Importantly, modern CT scanners achieve LIE imaging with only a minimal increase in radiation exposure compared to older systems (22). Multicenter collaboration can help overcome individual site limitations in patient volume, especially like MINOCA patients that may have lower incidence rates. Additionally, standardizing imaging protocols and data collection across centers will enhance the reliability and generalizability of the results, ultimately supporting wider clinical adoption of the findings. Larger, prospective studies are needed to confirm these findings and define the impact of this strategy on therapeutic decision-making and clinical outcomes.

## 5 Limitations

This retrospective two-center study included a relatively small number of participants, which limits the statistical power and the generalizability of the findings. With only 21 patients, it was not possible to calculate diagnostic accuracy metrics such as sensitivity, specificity, positive predictive value, or negative predictive value for LIE compared to LGE in detecting ischemic injury. Therefore, the current cohort is underpowered for reliable accuracy statistics. Imaging was performed within a standardized 14-day window, which, although practical, may have missed transient myocardial or coronary changes occurring in the very early stages of disease. The lack of a blinded core-laboratory analysis might have introduced some variability in interpretation across the different imaging modalities. Additionally, no formal assessment of inter- or intra-observer variability was conducted for LIE, which could affect the robustness and generalizability of the imaging results. The study did not use intracoronary imaging or functional testing, preventing a direct comparison with invasive reference standards (23). Extracellular volume fraction (ECV) quantification was not performed on the CCTA images, representing a limitation since ECV is an important marker of myocardial fibrosis and interstitial expansion. Future studies incorporating ECV measurement with CCTA, potentially with the help of emerging technologies like photon-counting detectors, may improve myocardial tissue characterization and overall diagnostic accuracy. Due to technical constraints and incomplete data, contrast-to-noise ratio (CNR) measurements were not available for all cases, limiting a comprehensive assessment. An additional important limitation relates to the difference in pharmacologic preparation between the CCTA and CMR protocols. In the CCTA protocol, patients received beta blockers (metoprolol) to control heart rate and sublingual nitroglycerin to optimize coronary artery caliber before image acquisition, whereas these medications were not administered in the CMR protocol. These interventions likely improved CCTA image quality by reducing motion artifacts and dilating the coronary arteries, enhancing lesion detection. CMR images, on the other hand, were acquired under physiological conditions without pharmacologic heart rate control or vasodilation, which may limit direct comparability of findings across modalities. It is important to recognize that this difference reflects established clinical practice for each imaging technique rather than a methodological flaw and should be considered when interpreting cross-modality comparisons. Finally, no long-term clinical outcome data were collected, preventing evaluation of the prognostic significance of the imaging findings. Given these limitations, this study should be viewed as a pilot investigation offering preliminary insights. Larger, prospective, multicenter studies with earlier imaging time points, standardized image evaluation, and longitudinal follow-up will be needed to confirm these findings and clarify their clinical relevance.

## 6 Conclusions

Early integration of coronary CT angiography (CCTA) and cardiovascular magnetic resonance (CMR) demonstrates promising potential to distinguish ischemic from non-ischemic myocardial injury in patients with suspected MINOCA. CCTA, complemented by quantitative plaque analysis and LIE, offers a unified anatomical and tissue characterization that parallels CMR findings while adding direct insight into coronary morphology. This multimodality approach may facilitate earlier etiological clarification, which could guide mechanism-based therapy and ultimately reduce adverse ischemic events. However, given the limited sample size, lack of independent validation, and absence of clinical outcome data, these findings should be interpreted cautiously. The present pivotal study does not establish diagnostic performance or prognostic impact but serves as a hypothesis-generating exploration into the utility of an integrated CCTA-CMR imaging protocol. Larger, prospective studies are required to confirm these preliminary observations, determine prognostic implications, and integrate this strategy into future management algorithms. Therefore, while a comprehensive CT protocol including LIE may represent a promising technique for rapid myocardial injury assessment in troponin-positive chest pain, further research is needed before firm clinical recommendations can be made.

## Data Availability

The original contributions presented in the study are included in the article/Supplementary Material, further inquiries can be directed to the corresponding author.
